# Identification of differentially expressed genes for *Pseudomonas* sp. Cr13 stimulated by hexavalent chromium

**DOI:** 10.1371/journal.pone.0272528

**Published:** 2022-08-05

**Authors:** Bingbing Pang, Hongling Yu, Jin Zhang, Fengcai Ye, Haifeng Wu, Changhua Shang

**Affiliations:** 1 College of Life Science, Guangxi Normal University, Guilin, China; 2 Key Laboratory of Ecology of Rare and Endangered Species and Environmental Protection (Guangxi Normal University), Ministry of Education, Guilin, China; 3 Guangxi Key Laboratory of Landscape Resources Conservation and Sustainable Utilization in Lijiang River Basin, Guangxi Normal University, Guilin, China; National Botanical Research Institute CSIR, INDIA

## Abstract

Over exploitation of mineral resources has increasingly caused serious heavy metal contamination such as chromium (Cr). Cr(VI), the pathogenicity factor, is one of common environmental contaminants and widely known health hazards to living organisms. Therefore, it is urgent to control the polluted soil. Up to now, little is known about the regulatory mechanisms of Cr response in *Pseudomonas* sp. Cr13. In this study, transcriptome and differentially expressed genes in *Pseudomonas* sp. Cr13 strain was characterized by a comparison between Cr(VI)-treated sample and control sample using transcriptome sequencing approach. In total, 2974 genes were annotated, including 1245 (1154 down-regulated genes and 91 up-regulated genes) differentially expressed genes (DEGs). All DEGs could be assigned to 29 pathways, of which pathways related to amino acid metabolism, carbohydrate metabolism, energy metabolism and signal transduction mechanism were significantly enriched in *Pseudomonas* sp. Cr13. A possible mechanism for Cr toxicity response might be an active efflux which utilized a heavy metal translocating P-type ATPase to lower the intracellular Cr concentration. The down-regulated genes related to the antioxidant defense system had a key role in Cr reduction, such as *SodA*, *Gst*, *osmC*, *BtuE*, *KatE*, *csdA* and *AhpC*. The proteins that were visibly up-regulated, were likely to involve in alleviating Cr(VI) stress, and the significantly down-regulated genes such as *MarR*, *Lrp*, *FhlA*, *GntR*, *HrcA*, *LysR* family genes, were likely to reduce Cr(VI) induced oxidative stress. In addition, real-time quantitative PCR was used to analyze the expression patterns of some Cr responsive genes. This study reported the first identification of Cr responsive genes, and inferred the underlying regulatory mechanisms of response to Cr(VI) stress in *Pseudomonas* sp. Cr13.

## 1. Introduction

Cr, the most abundant element in the earth’s crust, is considered as a non-essential element for plant nutrition and microorganisms [1, 2]. Cr is widely used and released into the environment by various industries [2, 3]. Cr(III) compounds have no adverse health effects on humans below a certain threshold [4–6]. However, Cr(VI) has been recognized as carcinogens and mutagens to human [7]. In addition, Cr(VI) was highly toxic metal, even 100 times highly carcinogenic than Cr(III) [8]. The common symptoms of Cr(VI) are as follows: gastro-enteritis, dermatitis, mucous and skin membrane ulcerations [5]. Long-term drinking of chromium-containing groundwater will cause hazard to the respiratory system [9]. What’s more, high concentration of chromium has an adverse effect on the physiological metabolism of plants [10, 11]. Therefore, the prevention and control of Cr(VI) contaminants in agricultural soil and water is crucial.

The remediation of Cr(VI) toxicity includes four categories: ecological methods, physical methods, chemical methods and biological methods. The biological methods are an important technology for soil remediation due to its advantages of low treatment cost, low environmental impact and high efficiency [12].

Phytoremediation by phytostabilization and phytoextraction had been increasingly attracting the concern of researchers [13]. Due to the well-developed root systems and high biomass in plants, srhizospheric microorganisms was able to remove certain Cr(VI) [14, 15]. Cr(VI) stress stimulated the expression of genes encoding reactive oxygen species scavenging enzymes, such as SOD and NADPH antioxidant enzyme. The components of glutathione-derived metal binding peptides, ATP-binding cassette transporters, phytochelatins and SOD were regarded as the important response mechanisms of oxidative stress to reduce oxidative damage caused by Cr [16, 17]. On the other hand, some studies had reported the molecular mechanism of Cr(VI) uptake, accumulation, translocation and detoxification in microorganisms. Microorganisms could convert toxic Cr(VI) to non-toxic Cr(III) by enzyme reduction or non-enzyme reduction to realize the detoxification of chromium [18]. *Pseudomonas umsongensis* CY-1 could effectively reduce Cr(VI) to less toxic Cr (III) [19]. Ectomycorrhizal fungi, which had a symbiotic relationship with plants, could reduce the toxicity of heavy metals in host plants. Its cell wall contained a mass of electronegative sites, so it had the ability to bind to Cr(VI) [15, 20]. Protein kinase CK2 and Neurospora crassa CHR-1 protein played an important role in counteracting heavy metal contaminants [21, 22]. The extracellular polymeric substance exopolysaccharide (EPS), secreted by *Pseudomonas aeruginosa*, is the major physiological mechanism of Cr(VI) reduction [23]. Although many studies have focused on the molecular and biochemical mechanisms of counteracting heavy metals in microorganisms, the information available is quite limited.

In the past, many species of *Pseudomonas* have been found to reduce Cr(VI) to Cr(III). Since 1970s, Cr(VI) reduction by *Pseudomonas* has been well recorded. Different species or strains of *Pseudomonas* have different adaptability to specific environment and different Cr(VI) reduction ability. *Pseudomonas* sp. Cr13 could effectively reduce Cr(VI) in soil, which laid a good foundation for the bioremediation of heavy metal Cr(VI) in Dachang town, Hechi city of Guangxi autonomous region.

In previous study, we isolated a strain Cr13 with significant Cr(VI) tolerance and Cr(VI) removal ability from mine-contaminated soils in Dachang town, Hechi city of Guangxi autonomous region, and this strain was identified as *Pseudomonas* genus by 16S rDNA sequencing [24]. We also used Biolog GEN III MicroStation to further identify this strain, but there was no result. Therefore, we thought that it might be a new species. We will further identify this strain using other experiments in the future. However, very little is known about the molecular mechanisms of Cr(VI) tolerance and Cr(VI) removal in *Pseudomonas* sp. Cr13.

Transcriptomic technology was widely carried out on functional gene identification and transcriptome analysis. Recently, *Pseudomonas* sp. Cr13 with high Cr(VI) resistance and high Cr(VI) removal efficiency was isolated, but the underlying molecular mechanisms of Cr tolerance have not yet been reported in *Pseudomonas* sp. Cr13. In the paper, Illumina sequencing was used for a comparison between control group and Cr(VI) treated group in order to reveal the potential genes related to Cr tolerance. The purpose was to explore a deeper mechanism of response to Cr stress in strain Cr13 and reveal the key genes related to Cr(VI) tolerance. The results in this paper would lay a good foundation for the bioremediation of heavy metals pollution in the future.

## 2. Experimental section

### 2.1. Heavy metal treatment

Our laboratory had isolated a *Pseudomonas* sp. Cr13 strain with high Cr(VI) removal efficiency from Dachang town, Nandan county, Hechi city of Guangxi autonomous region [24]. LB medium (pH 7.0) was used in this study, which contained 10 g/L tryptone, 10 g/L NaCl, 5 g/L yeast extract [25]. *Pseudomonas* sp. Cr13 strain was inoculated into LB liquid medium (control sample, CrC) and LB liquid medium with 100 mg/L Cr(VI) (treated sample, CrT), respectively. Because strain Cr13 had better growth under a moderate Cr(VI) concentration (100 mg/L, originated from K_2_Cr_2_O_7_), therefore Cr(VI) was added into LB medium at the beginning. For RNA extraction, two samples were incubated at 30°C with 180 rpm for 16 h.

### 2.2. Total RNA extraction and quality assessment

2 mL of bacterial culture was centrifuged at 12000 rpm for 3 min. Total RNA was extracted from two samples (CrC and CrT) using Total RNA Extractor (Trizol) (Sangon Biotech, China). To ensure that enough qualified samples were used for transcriptome sequencing, RNA detection involved in the following two methods. RNA samples were detected for degradation and contamination using 1% agarose gel. RNA purity and concentration were determined by Qubit 3 Fluorometer (Thermo Fisher, USA). Total RNAs were extracted from two samples (CrC and CrT) in equal amounts for cDNA library construction.

### 2.3. Library construction and RNA-seq

The library was constructed with high-quality RNA samples using VAHTS Stranded mRNA-seq Library Prep Kit for Illumina (Vazyme, China). The mRNA was enriched and purified using mRNA Capture Beads, and then randomly fragmented into short fragments. Using short fragment as template, first-strand cDNA was synthesized with random hexamer primer and 1st Strand Enzyme Mix. The 2nd strand cDNA was then synthesized with 2nd Strand/End Repair Enzyme Mix. After the purification of cDNA with VAHTS DNA Clean Beads, dA-tail and adapter were added into cDNA. The suitable fragments were obtained for PCR amplification. Qubit 3 Fluorometer was used to evaluate the library concentration. Finally, cDNA library was sequenced using Illumina HiSeq 2000 platform. The clean reads were assembled to generate contigs.

### 2.4. Data assembly and functional annotation

Data assembly and functional annotation were performed based on our former study [26]. In order to get clean reads, according to the Phred quality score of original data (raw reads) and other information, FastQC (version 0.11.2) was used to evaluate the quality of sequencing data. Raw reads often contain low quality sequences. In order to ensure the quality of information analysis, raw reads with adaptors and unknown nucleotides, overall low-quality reads were removed using Trimmomatic software (version 0.36) to generate clean reads. Subsequently, high-quality sequencing datas (clean reads) were assembled by Rockhopper (version 2.0.3) which was a novel software for assembly, and specific to bacterial high-throughput transcriptome sequencing [27]. After the assembly, all unigene sequences were aligned against CDD, COG, NR, NT, PFAM, SWISS-PROT, TrEMBL and other databases to obtain functional annotation. Go annotation was obtained by aligning the unigenes against SWISS-PROT and TrEMBL. KEGG annotation of unigenes was obtained by KAAS. CDS prediction was performed based on Transdecoder. KEGG pathway and COG were analyzed using Clusterprofiler. Unigene sequences were compared with NCBI non-redundant proteins using BLAST (http://ncbi.nlm.nih.gov/). SWISS-PROT was a manually annotated and protein knowledgebase, but TrEMBL was an automated annotation (http://www.ebi.ac.uk/uniprot) [28].

### 2.5. Gene expression analysis

The direct embodiment of gene expression level is the abundance of its transcript. The transcript abundance is higher, gene expression level is higher. we can estimate gene expression level in RNA-seq analysis by the count of sequencing reads located to genomic regions or exon regions. As a common method, TPM was introduced to estimate gene expression level, which considered the sequencing depth, gene length and the effect of samples on count.

### 2.6. KEGG, COG and GO enrichment analysis

The purpose of gene enrichment analysis is to find out the gene sets with different expression levels. Gene enrichment analysis developed from expression difference analysis of single gene to expression difference analysis of gene sets. Enrichment analysis improves the reliability of the study. Enrichment analysis can identify the most relevant biological pathway. Use clusterProfiler for functional enrichment analysis because GO is a directed acyclic structure, use topGO alone for GO enrichment. Normally, when the corrected *P* value less than 0.05, it is considered that there is a significant enrichment of the function.

### 2.7. Quantitative real-time PCR (qRT-PCR)

To confirm the reliability of transcriptome sequencing, we randomly selected three candidate genes to preform qRT-PCR. Gene specific primers and 16S rDNA gene primers were designed using Beacon Designer 7.0 software. About 2 μg of total RNA (CrC and CrT) was extracted. Then cDNA was synthesized by reverse transcriptase MMLV, and was used as the template (final concentration 50 ng/μL-100 ng/μL) for qRT-PCR. qRT-PCR was performed using a 2xSYBR Green qPCR Mix Kit with the following procedure (94°C 15 s, 60°C 30 s, 40 cycles). The experiments were achieved in triplicate.

## 3. Results

### 3.1. Assembly of the transcriptome data

A total of 24118498 and 48695670 clean reads were obtained from two samples CrC and CrT (Table 1). The bases proportions of Q10, Q20, Q30 were 99.54%, 96.98%, 91.26% in CrC and 99.60%, 98.16%, 92.20% in CrT. Using Rockhopper software, 2974 unigenes were generated with average length of 1351.91bp (Table 2). N50 is one of the important indexes to evaluate the assembly quality. It is usually considered that N50>1000 bp represents the better assembly quality. The results (N50 = 1955, Table 2) indicated that the data had high quality.

**Table 1:** Sequencing data for CrC and CrT.

	CrC	CrT
Total reads count	48695670	24118498
Total bases count (bp)	6997455330	3454059536
Average read length (bp)	143.7	143.21
Q10 bases count (bp)	6965121606	3440370132
Q10 bases ratio	99.54%	99.60%
Q20 bases count (bp)	6847617416	3390346573
Q20 bases ratio	97.86%	98.16%
Q30 bases count (bp)	6386089295	3184483257
Q30 bases ratio	91.26%	92.20%
N bases count (bp)	1257717	634438
N bases ratio	0.02%	0.02%
GC bases count (bp)	3963412474	1947713710
GC bases ratio	56.64%	56.39%

**Table 2:** Assembled unigenes from transcriptome analysis.

Count	> = 500 bp	> = 1000 bp	N50	N90	Average length
2974	2253	1477	1955	702	1351.91

### 3.2. Functional annotation and classification

To obtain the functional annotation information, the unigenes were compared with nine databases (CDD, COG, NR, NT, PFAM, SWISS-PROT, TrEMBL, GO, KEGG). In total, 2974 genes were annotated as follows: 2338 genes in CDD (78.61%), 2108 genes in COG (70.88%), 2855 genes in NR (96%), 2578 genes in NT (86.88%), 1898 genes in PFAM (63.82%), 2196 genes in SWISS-PROT (73.84%), 2849 genes in TrEMBL (85.8%), 2424 genes in GO (81.51%), and 815 genes in KEGG (27.4%). Among these annotated unigenes, 2883 unigenes were annotated in at least one database (96.94%) and 690 unigenes were annotated in all databases (23.2%).

**Fig 1:**
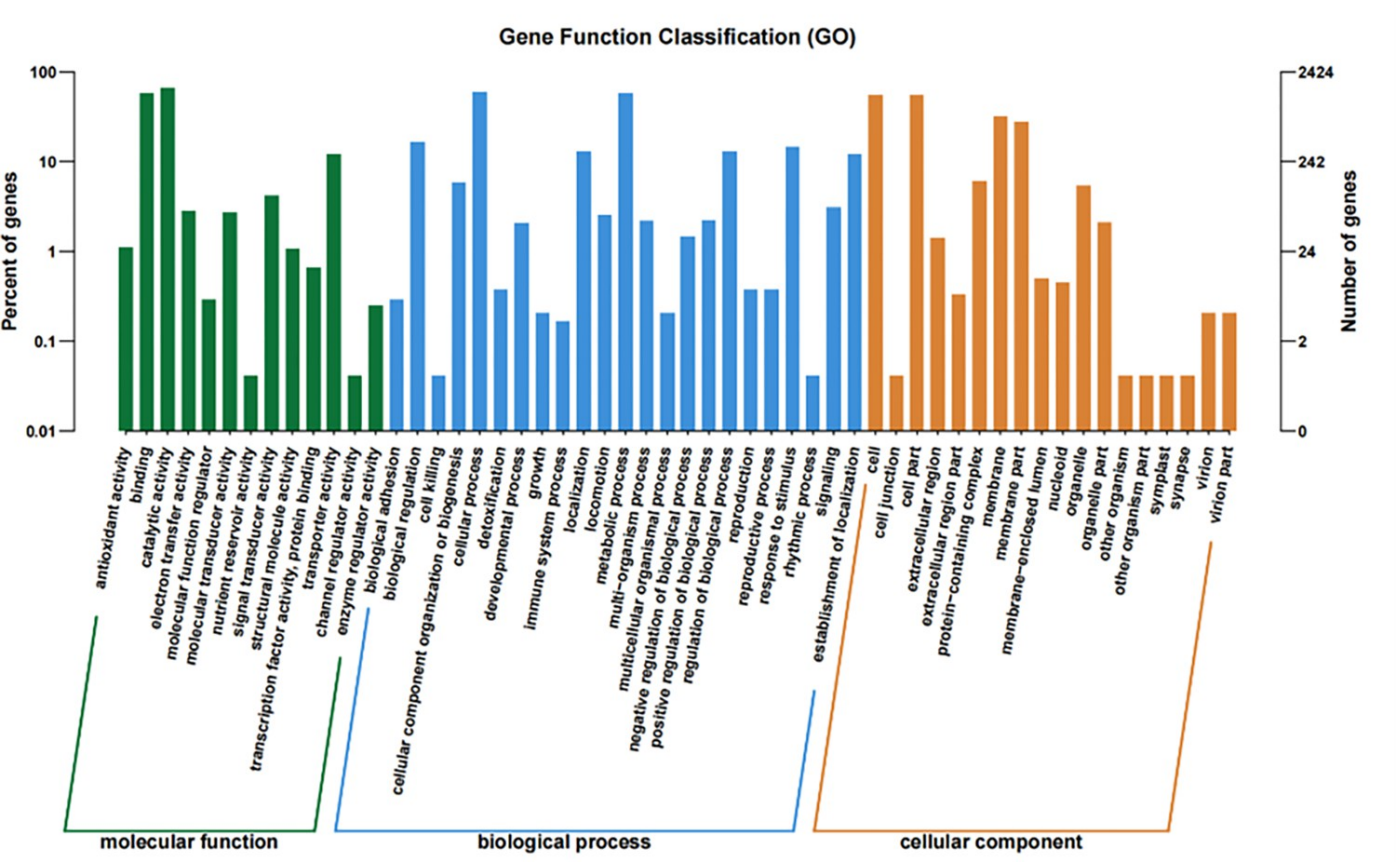
GO enrichment analysis of unigenes. The X-axis showed GO function. The right Y-axis showed number of genes, and the left Y-axis showed the percentage.

For GO classification, all unigenes were classified into three categories, including molecular functions, biological process, and cellular component (Fig 1). Among various categories of molecular function, binding, catalytic activity and transporter activity were the most abundant categories. In the classification of biological process, the largest categories included biological regulation, cellular process and metabolic process. In the classification of cellular component, three dominant categories were cell, cell part and membrane (Fig 1).

**Fig 2:**
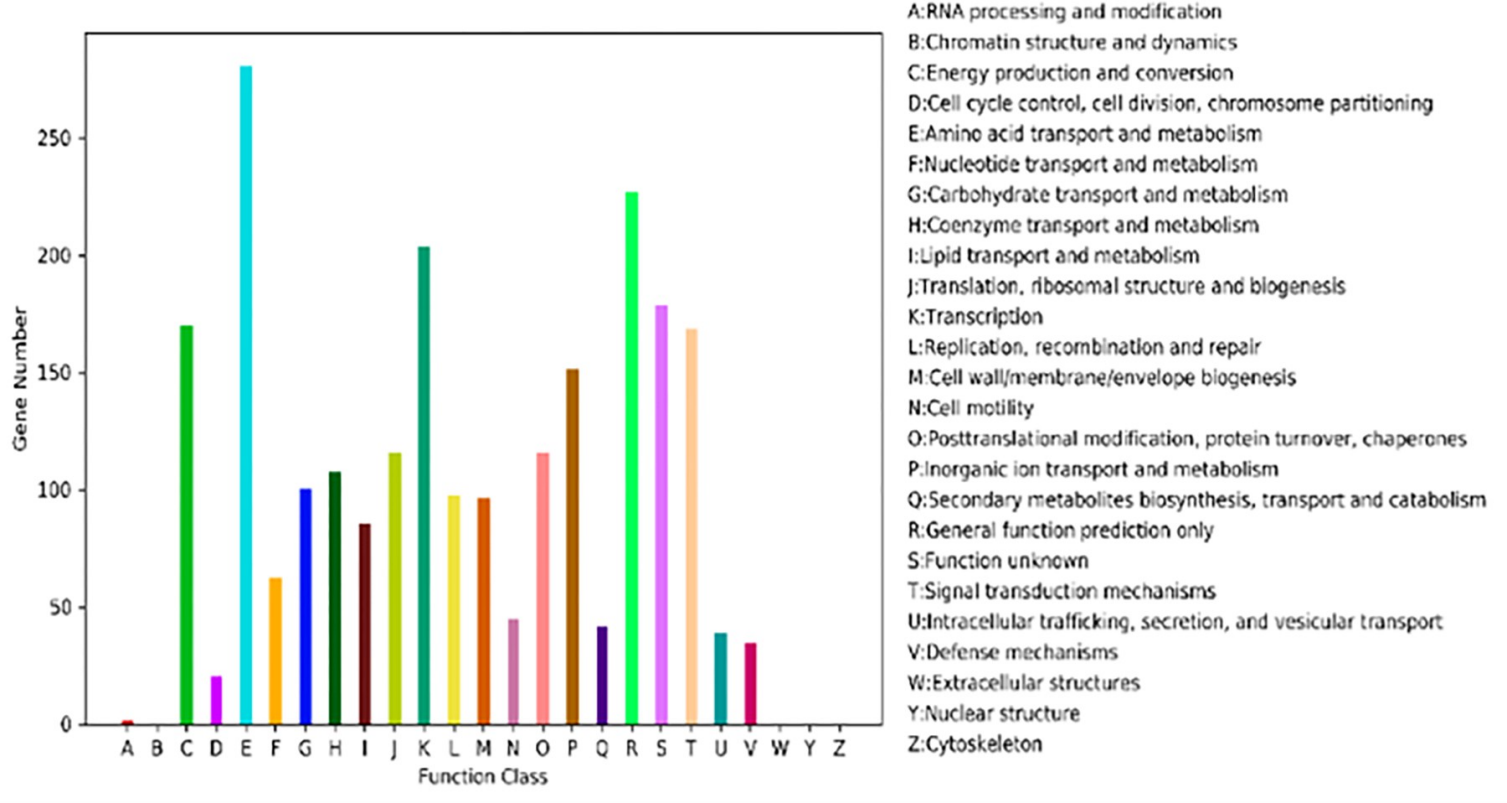
COG enrichment analysis of unigenes.

A total of 2351 unigenes were classified in COG database. Among them, the dominant functional classification contained energy production and conversion, amino acid transport and metabolism, transcription, inorganic ion transport and metabolism, general function prediction only and signal transduction mechanisms. These unigenes related to inorganic ion transport and metabolism, may participate in the respond to heavy metal stress in Cr13 strain (Fig 2). Three kinds of major significantly up-regulated genes coded for proteins related to energy production and conversion, signal transduction mechanism, amino acid transport and metabolism (Table 3). The down-regulated genes related to the antioxidant defense system had an important role in Cr reduction. Glutathione S-transferase, thioredoxin, Glutathione peroxidase responded strongly to Cr stress. The Cr stress response also focused on reducing the intracellular metal concentration with multiple ABC-type transport system, while a transporter gene was down-regulated to reduce uptake of Cr (Table 4).

**Table 3:** Selected up-regulated genes under Cr(VI) stress in strain Cr13.

Functional category	Gene annotation	log_2_Fold change
Inorganic ion transport and metabolism		
*IrpA*	Uncharacterized iron-regulated protein	3.16
*HemO*	Heme oxygenase	3.28
*AfuA*	ABC-type Fe^3+^ transport system	3.08
*FecR*	Fe^2+^ dicitrate sensor, membrane component	1.35
*TauA*, *TauC*	ABC-type nitrate, sulfonate, bicarbonate transport systems	1.26, 1.16
*FecA*	Outer membrane receptor for Fe^3+^ dicitrate	1.75
*Bfd*	Bacterioferritin associated ferredoxin	2.08
Lipid metabolism		
*AtoA*, *Acyl*	Acetate/3-ketoacid CoA transferase, beta subunit	2.23
*OLE1*	Fatty-acid desaturase	2.31
Energy production and conversion		
*CydA*	Cytochrome bd-type quinol oxidase	1.07
*CaiB*	Acyl-CoA transferases/carnitine dehydratase	3.06
*LdhA*	Lactate dehydrogenase	7.684
Defense mechanisms		
*AcrB*	Cation/multidrug efflux pump	1.67
*TolC*	Outer membrane protein	1.93
Amino acid transport and metabolism		
*BetA*	Choline dehydrogenase and related flavoproteins	1.54
*GlnQ*	ABC-type polar amino acid transport system, ATPase component	1.88
*SerA*	Phosphoglycerate dehydrogenase and related dehydrogenases	1.01
*AnsB*	L-asparaginase/ archaeal Glu-tRNAGln amidotransferase subunit D	1.209
*Asl*	Argininosuccinate lyase	3.53
*GabT*	4-aminobutyrate aminotransferase and related aminotransferases	1.16
*glyA*	Glycine/serine hydroxymethyltransferase	1.38
Signal transduction mechanisms		
*Crp*	cAMP-binding domains-Catabolite gene activator and regulatory subunit of cAMP-dependent protein kinases	4.96
*FecR*	Fe^2+^ dicitrate sensor, membrane component	1.35
DNA replication, recombination, and repair		
*Exo*	5’-3’ exonuclease (including N-terminal domain of PolI)	2.61

**Table 4:** Selected down-regulated genes under Cr(VI) stress in strain Cr13.

Functional category	Gene annotation	log_2_Fold change
Transcription		
*MarR*, *Lrp*, *HrcA FhlA*, *GntR*, *LysR*	Transcriptional regulators	-3.02, -1.70, -19.17,-1.50, -3.02, -4.18
*XylA*	Xylose isomerase	-18.62
Carbohydrate transport and metabolism		
*MalK*	ABC type sugar transport systems, ATPase components	-22.58
*ZntA*, *MgtA*	Cation transport ATPase	-3.23, -1.48
*CopZ*	Copper chaperone	-2.04
Oxidative stress		
*Gst*,	Glutathione S-transferase	-1.02
*BtuE*	Glutathione peroxidase	-9.40
*osmC*	Organic hydroperoxide reductase	-1.06
*KatE*	Catalase	-1.07
*csdA*	Cysteine desulfurase	-12.14
*AhpC*	Peroxiredoxin	-2.09
*SodA*	Superoxide dismutase	-20.57

**Fig 3:**
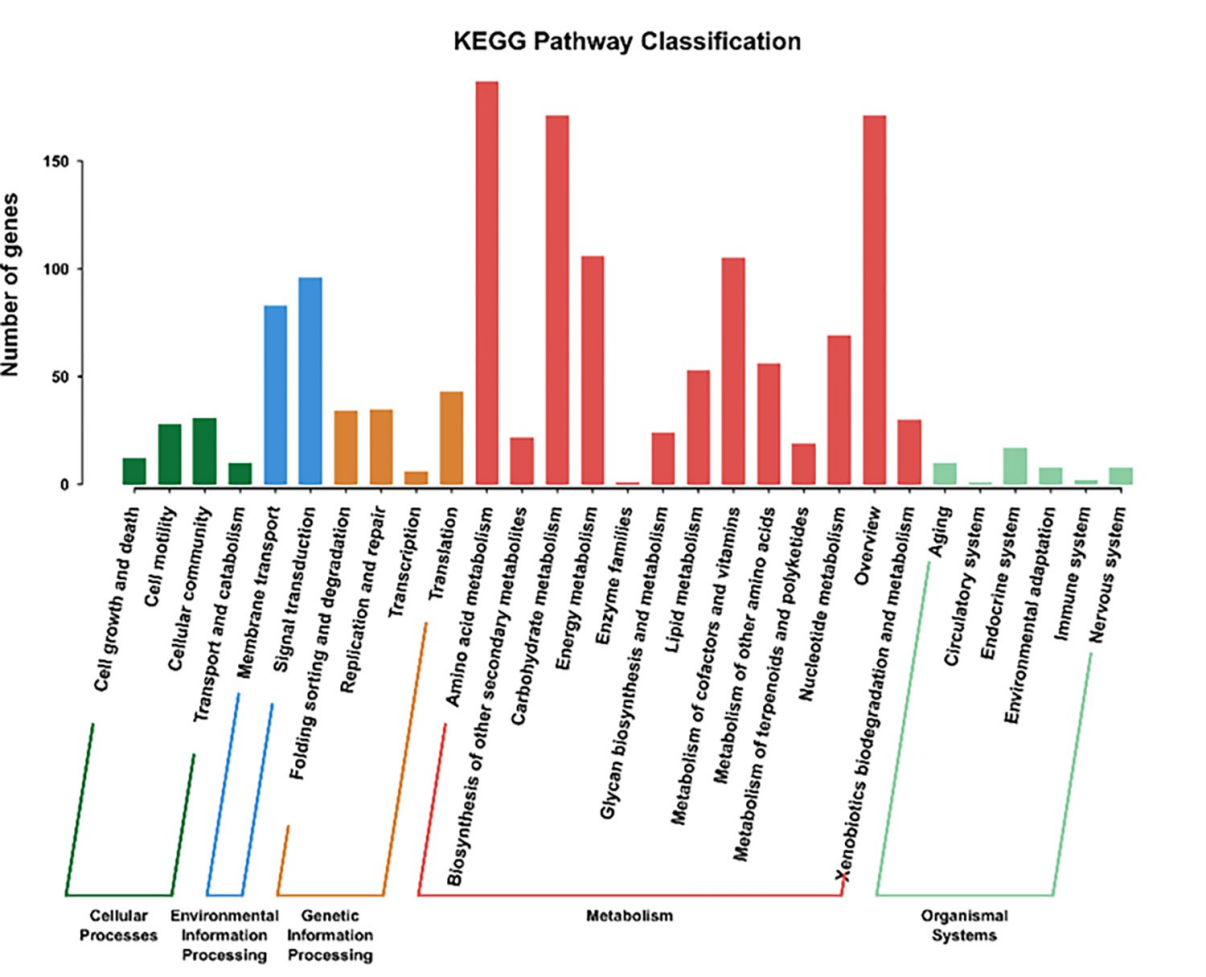
KEGG metabolic pathways. X-axis indicates different KEGG categories and Y-axis indicates the number of genes related to KEGG pathway.

1438 unigenes were classified into 29 metabolic pathways, and all KEGG pathways were displayed in Fig 3. There were four pathways involved in cellular process. Two pathways were associated with environmental information processing, four pathways were related to genetic information processing, and 13 pathways were related to metabolism. At last, there were 6 pathways related to organic system. Among 29 metabolic pathways, amino acid metabolism, carbohydrate metabolism, energy metabolism, cofactor and vitamin metabolism were four dominant pathways, which were the important metabolic pathways of primary metabolism and were closely related to the growth and development of organisms. To date, though, the molecular mechanisms of resistance to Cr toxicity is not clear, genes with different expression levels can explain the molecular mechanism by annotation.

### 3.3. Differentially expressed genes of Cr13 strain under Cr(VI) stress

In order to identify genes in response to Cr(VI) stress. *Pseudomonas* sp. Cr13 strain was inoculated into LB medium with Cr(VI) (100 mg/L, CrT) and LB medium without Cr(VI) (control sample, CrC). Then RNA was extracted for RNA-seq. To get significantly different genes, we used DESeq for analysis. A total of 1245 genes were differentially expressed genes (DEGs). Among DEGs, 91 genes were up-regulated and 1154 genes were down-regulated. A ferredoxin gene *Bfd* was 2.08-fold up-regulated following Cr(VI) stress based on RNA-Seq. In previous studies, bacteria relied on a well-regulated iron homeostasis to survive in adverse environments. An important component of iron homeostasis machinery was the compartmentalization of Fe^3+^ in bacterioferritin (BfrB) and its subsequent mobilization as Fe^2+^ by binding of a ferredoxin (Bfd) to satisfy metabolic requirements [29, 30]. In addition, the BfrB-Bfd complex was blocked by the deletion of *Bfd* gene, which resulted in an irreversible accumulation of iron in BfrB, and affected central carbon metabolism and amino acid biosynthesis [29, 30]. This indicated that ferredoxin probably affected Cr(VI) reduction process in strain Cr13. On the other hand, *CydA* encoding cytochrome bd-type quinol oxidase, played an important role in the survival of *Mycobacterium smegmatis* under antibiotic induced peroxide stress [31]. In previous study, the treatment of NaN_3_ (a cytochrome inhibitor) resulted in significant decrease of Cr(VI) reduction ability, which indicated that cytochrome were involved in Cr(VI) reduction in strain LZ01 [32]. In *Desulfovibrio vulgaris*, cytochrome C3 as a reductase could reduce toxic and highly soluble Cr(VI) to less toxic and less soluble Cr(III), and reduce the soluble oxidized form of uranium (U^6+^) from contaminated water to insoluble U^4+^ when cytochrome C3 was combined with pure periplasmic hydrogenase. These results demonstrated that cytochrome C3 was involved in electron transport in whole cells, and hydrogenase was the physiological electron donor for cytochrome C3 [33]. Besides, c-type cytochromes, as an intermediary electron transporter, were involved in Cr(VI) reduction. It was suggested that cytochrome system was related to Cr(VI) extracellular reduction [34]. Previous study had also reported that heavy metal transporters played an important role in metal detoxification, resistance and homeostasis in bacteria [35]. Genes coding for inorganic ion transport and metabolism, such as *AfuA* (1.5-fold) and *TauA* (3.5-fold), were all up-regulated under Cr stress. Gene *AcrB* coding for a cation/multidrug efflux pump, was up-regulated (1.67-fold). Cation efflux family were associated with the detoxification of Cr (Table 3). In *Caulobacter crescentus*, the transcripts of two clusters of efflux pump genes were specifically up-regulated under heavy metal stress [36]. Other genes (*Gst*, *osmC*, *BtuE*, *KatE*, *csdA*, *AhpC* and *SodA*) related to antioxidant defense system and removal of toxic compounds were down-regulated (Table 4). Previous study also reported that glutathione S-transferase detoxified the products of oxidative stress by the covalent linking of hydrophobic substrates to glutathione [37]. *SodA*, an enzyme, showed about 20-fold down-regulation in *Pseudomonas* sp. Cr13 under Cr(VI) stress. Superoxide dismutase (SOD) can catalyze the conversion of superoxide radical to hydrogen peroxide and molecular oxygen, and hydrogen peroxide can be further detoxified to protect bacteria from xenobiotic [38]. Our data indicated that *MarR*, *Lrp*, *FhlA*, *GntR*, *HrcA*, *LysR* family were involved in the response to Cr. In previous research, a *MarR* family transcriptional regulator was down-regulated in response to Cr exposure in *Shewanalla oneidensis* MR-1 [39]. Our data also showed that the genes (*MalK*, *ZntA*, *MgtA*, *CopZ*) were all down-regulated (more than 2.0-fold) under Cr stress. A possible mechanism for Cr toxicity response might be an active efflux utilizing a translocating P-type ATPase to lower the intracellular Cr concentration in *Pseudomonas* sp. Cr13.

### 3.4. KEGG pathway enrichment of DEGs

Among up-regulated genes, 61 genes were categorized into transport and binding proteins, and 16 genes were associated with regulatory function. Only six genes were significantly enriched in transcription pathway, and genes coding for transcription regulator (*MarR*, *Lrp*, *FhlA*, *GntR*, *HrcA*, *LysR* family) were significantly down-regulated in *Pseudomonas* sp. Cr13. In KEGG pathways, inorganic ion transport and metabolism, ABC type transport system, lactoylglutathione lyase, resistance protein ARSH, CusA/CzcA family heavy metal efflux RND transporter, peroxidase (GSH, CAT, SOD), siderophore biosynthesis protein, coenzyme A disulfide reductase were associated with down-regulated genes. Whereas, chromate efflux transporter was associated with up-regulated genes. Fatty acid oxidation and ether phospholipid biosynthesis mainly depended on peroxidase, which played an important role in coordinating abiotic stresses and detoxifying xenobiotic chemicals. CAT and SOD, from hydrogen peroxide and glutathione metabolism, could remove reactive oxygen free radicals and protect the organism from oxidative damage [40, 41]. SOD can catalyze the conversion of superoxide radicals to hydrogen peroxide and molecular oxygen, which can be further detoxified to protect the bacteria from xenobiotic [38]. This experiment proved that SOD activity was significantly inhibited in the presence of Cr(VI). In addition, CAT activity was also inhibited when *Pseudomonas* sp. Cr13 was permanently exposed to high concentration of Cr(VI), which led to an increasing amount of H_2_O_2_ and disturbed the antioxidant defense system. Therefore, the detoxification mechanism of *Pseudomonas* sp. Cr13 was evaluated by changes in CAT and SOD activities.

### 3.5. qRT-PCR validation

**Fig 4:**
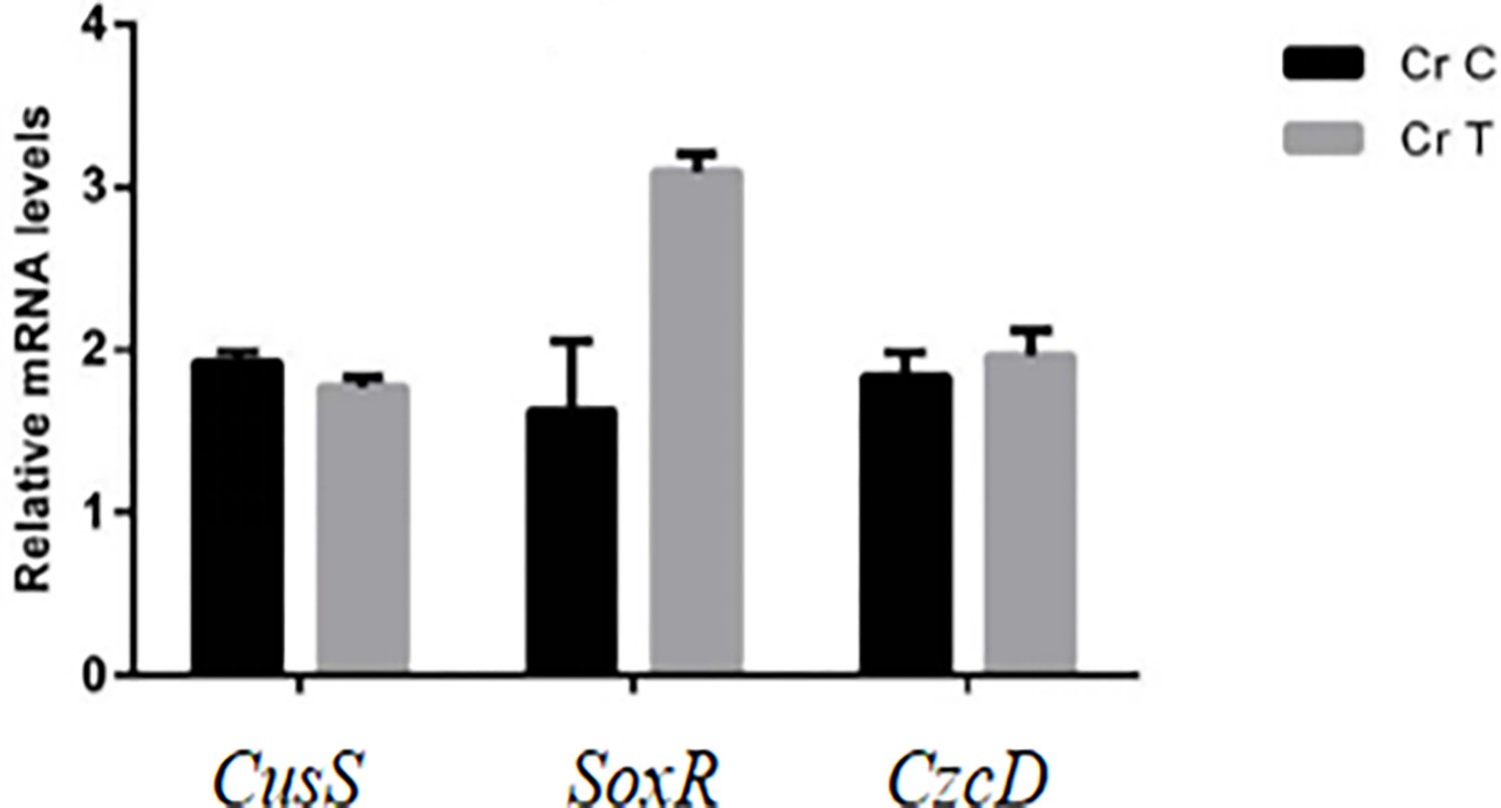
qRT-PCR results of three randomly selected candidate genes. The 16S rRNA gene was used as an internal reference. The data was shown as mean ± standard error (n = 3).

To verify the reliability of RNA-seq analysis, three candidate genes were selected for qRT-PCR validation at 16 h after Cr(VI) treatment, which included *CusS* gene related to heavy metal sensor histidine kinase, *SoxR* family gene and *CzcD* gene associated with heavy metal ion efflux system component. Compared with the control group, all genes were differentially expressed. The expression of *cusS* gene was significantly down-regulated and the member of SoxR family was significantly up-regulated under the treatment of 100 mg/L Cr(VI) compared with the control (Fig 4). The SoxR family gene encodes a transcriptional regulator, an important nuclear protein involved in the regulation of gene expression. When *Pseudomonas* sp. Cr13 were exposed to Cr(VI) stress, which activates or deactivates the expression of target genes in large quantities by losing an electron in the iron sulfur group. A great number of studies indicated that *Pseudomonas aeruginosa* SoxR could activate six gene regulators in the oxidative stress response, and as a regulatory factor, SoxR directly involved in quorum sensing of *P*. *aeruginosa* [42, 43]. Therefore, SoxR can be used as an index to estimate the stress state in *Pseudomonas* sp. Cr13.

## 4. Discussion

The *Pseudomonas* genus, Gram negative bacteria, are widely distributed in the nature, and the strain *Pseudomonas* sp. Cr13 can resist high concentration of Cr^6+^ (up to 250 mg/L) and Cd^2+^ (50 mg/L) [24]. Numerous studies have shown that *Pseudomonas* significantly alleviated Cr^6+^ toxicity and enhanced the growth and nutrient uptake of plant as bionic bacteria [44]. Several studies have reported that extracellular reduction (75% removal rate) and adsorption by cell wall (24% removal rate) were the main physiological mechanisms of Cr(VI) removal [45, 46]. However, the metabolic mechanism and cellular response have not been explored in *Pseudomonas* sp. Cr13. In this research, cDNA library was constructed from the control group and treatment group treated with Cr^6+^ in order to assess gene expression levels in response to Cr^6+^. In the presence of Cr(VI), the obviously down-regulated unigenes were mainly related to amino acid transport, transport and metabolism of inorganic ions and ATPase transport. The results suggested that Cr(VI) had a significant influence on the antioxidant system of *Pseudomonas* sp. Cr13. In addition, in *Pseudomonas* sp. Cr13, some molecular and physiological mechanisms of the above phenomenon could be explained by *CusS* gene. For example, in *P*. *aeruginosa*, the CzcR-CzcS two-component system primarily participated in metal detoxification and antibiotic resistance through intracellular regulatory pathway [47]. Recent studies had revealed that *CusS* involved in metal ions transport, and *CusS* stimulated the phosphorylation of the outer membrane protein in the presence of heavy metals. The efflux pump gene *CusCFBA* was highly expressed, which pumped copper ion out of the bacteria. Therefore, two-component system heavy metal sensor histidine kinase can promote bacteria to relieve oxidative stress [48].

SOD activity was significantly inhibited in the presence of Cr(VI). SOD can catalyze the conversion of superoxide radicals to hydrogen peroxide and molecular oxygen, which can be further detoxified to protect the bacteria from xenobiotic chemicals inside the cell. In addition, CAT activity was also be inhibited under high concentration of Cr(VI), which led to an increasingly amount of H_2_O_2_ and disturbed the antioxidant defense system.

In *Pseudomonas* sp. Cr13, greatly down-regulated unigenes were mainly associated with *MarR* family, ABC transporters, Tolc family proteins, metal ions resistance protein *ARSH*, and signal-regulated kinases. Meantime, significantly up-regulated unigenes were primarily related to efflux transporter and DNA binding transcription factor. Furthermore, as an important up-regulated unigene, *MerR* plays an important role in against oxidative damage, the expression of MerR mRNA was significantly higher than other genes in *Pseudomonas* sp. Cr13. Numerous studies have revealed that MerR family genes greatly alleviated oxidative stress in bacteria by Cd(II)/Pb(II)-responsive transcriptional regulator. For example, MerR family genes involved in detoxification in bacterial systems under oxidative stress caused by metal ions. *CadR* was closely related to MerR family of mercury stress regulator, which positively promote the expression of mercury-detoxifying genes to relieve heavy metal stress [49]. In addition, in *Pseudomonas putida* PNL-MK25, *cueAR* belongs to MerR family, and consists of two genes, which encode a MerR-type metalloregulatory protein (CueR), and a putative P1-type ATPase (CueA), respectively. CueAR played an obvious role in copper homeostasis and conferred protection against oxidative damage [50].

Therefore, we speculated that enzymes such as SOD and CAT could relieve oxidative stress, and prevent cells from oxidative damage by inorganic ion transport and metabolism. What’s more, *CusS* gene accelerated extracellular reduction and cell wall adsorption, and MerR family genes relieved Cr(VI) damage by Cd(II)/Pb(II)-responsive transcriptional regulator to help *Pseudomonas* sp. Cr13 against oxidative damage.

In summary, we presented a transcriptomic analysis of *Pseudomonas* sp. Cr13 under Cr(VI) stress using Illumina sequencing. The transcripts were annotated by nine databases (CDD, COG, NR, NT, PFAM, SWISS-PROT, TREMBL, GO, KEGG). In total, 2974 genes were annotated between 100 mg/L Cr(VI) treated sample and control group, of which 1245 DEG were annotated, including 91 up-regulated genes and 1154 down-regulated genes, respectively. The transcriptomic response provided important information for studying related genes and regulatory mechanisms in Cr(VI)-tolerant strain towards Cr(VI) stress. The findings herein indicated that the antioxidant system suffered from obvious effects under Cr(VI) stress. This system significantly involved in Cr(VI) reduction to alleviate Cr(VI) stress. For example, SOD can catalyze the conversion of superoxide radicals to hydrogen peroxide and molecular oxygen, which can be further detoxified to protect the bacteria from xenobiotic chemicals inside the cell. Furthermore, CAT activity was also inhibited under high concentration of Cr(VI), which led to an increasingly amount of H_2_O_2_ and disturbed the antioxidant defense system. In addition, KEGG pathway enrichment analysis suggested that the visibly up-regulated genes related to the hypothetical protein, were likely to have a hand in alleviating Cr(VI) stress in *Pseudomonas* sp. Cr13. The significantly down-regulated transcriptional regulators (*MarR*, *Lrp*, *FhlA*, *GntR*, *HrcA*, *LysR* family) were also likely to reduce Cr(VI) induced oxidative stress. The qRT-PCR analysis revealed that DEGs related to antioxidant system played an important role in Cr(VI)-tolerant strain towards Cr(VI) stress. In conclusion, peroxidase, amino acid synthesis, inorganic ion transport and metabolism, ABC type transport system, lactoylglutathione lyase, siderophore biosynthesis protein or defense-related protein were the effective metabolic pathways to counteract the toxicity of Cr. Overall, our findings contributed significantly to understanding of specific gene related to the antioxidant defense system and Cr detoxification in Cr(VI)-tolerant strain. In addtion, this results will enable us to take efficient methods to counteract the toxicity of Cr in soil containing heavy metals as well as to reduce Cr uptake in host plants.

## Supporting information

S1 Table Differentially expressed genes under Cr(VI) stress in strain Cr13. (XLS)

## References

[1] Liu D., Zou J., Wang M., Jiang W., Hexavalent chromium uptake and its effects on mineral uptake, antioxidant defence system and photosynthesis in *Amaranthus viridis* L., Bioresour. Technol. 99 (2008) 2628–2636. 10.1016/j.biortech.2007.04.045 17570658

[2] Shanker A.K., Djanaguiraman M., Venkateswarlu B., Chromium interactions in plants: current status and future strategies, Metallomics. 1 (2009) 375–383. doi: 10.1039/b904571f 21305140

[3] Awasthi Y., Ratn A., Prasad R., Kumar M., Trivedi A., Shukla J.P., et al., A protective study of curcumin associated with Cr^6+^ induced oxidative stress, genetic damage, transcription of genes related to apoptosis and histopathology of fish, *Channa punctatus* (Bloch, 1793), Environ. Toxicol. Pharmacol. 71 (2019) 103209. 10.1016/j.etap.2019.103209 31207396

[4] Li S.G., Hou J., Liu X.H., Cui B.S., Bai J.H., Morphological and transcriptional responses of *Lycopersicon esculentum* to hexavalent chromium in agricultural soil, Environ. Toxicol. Chem. 35 (2016) 1751–1758. 10.1002/etc.3315 26627465

[5] Baruthio F., Toxic effects of chromium and its compounds, Biol. Trace Elem. Res. 32 (1992) 145–153. doi: 10.1007/BF02784599 1375051

[6] Vincent J.B., Effects of chromium supplementation on body composition, human and animal health, and insulin and glucose metabolism, Curr. Opin. Clin. Nutr. Metab. Care. 22 (2019) 483–489. doi: 10.1097/MCO.0000000000000604 31577642

[7] Viti C., Marchi E., Decorosi F., Giovannetti L., Molecular mechanisms of Cr(VI) resistance in bacteria and fungi, FEMS Microbiol. Rev. 38 (2014) 633–659. doi: 10.1111/1574-6976.12051 24188101

[8] García-Hernández M.A., Villarreal-Chiu J.F., Garza-González M.T., Metallophilic fungi research: an alternative for its use in the bioremediation of hexavalent chromium, Int. J. Environ. Sci. Technol. 14 (2017) 2023–2038.

[9] Herrmann M.S., Testing the waters for chromium, J. Chem. Educ. 71 (1994) 323–324.

[10] Shewry P.R., Peterson P.J., The uptake and transport of chromium by barley seedlings (*Hordeum vulgare* L.), J. Exp. Bot. 25 (1974) 785–797.

[11] Panda S., Chaudhury I., Khan M., Heavy metals induce lipid peroxidation and affect antioxidants in wheat leaves, Biologia. Plantarum. 46 (2003) 289–294.

[12] Srivastava N.K., Majumder C.B., Novel biofiltration methods for the treatment of heavy metals from industrial wastewater, J. Hazard Mater. 151 (2008) 1–8. doi: 10.1016/j.jhazmat.2007.09.101 17997034

[13] Pilon-Smits E., Phytoremediation, Annu. Rev. Plant Biol. 56 (2005) 15–39. doi: 10.1146/annurev.arplant.56.032604.144214 15862088

[14] Velez P.A., Talano M.A., Paisio C.E., Agostini E., González P.S., Synergistic effect of chickpea plants and *Mesorhizobium* as a natural system for chromium phytoremediation, Environ. Technol. 38 (2017) 2164–2172. doi: 10.1080/09593330.2016.1247198 27788623

[15] Shi L., Deng X., Yang Y., Jia Q., Wang C., Shen Z., et al., A Cr(VI)-tolerant strain, *Pisolithus* sp1, with a high accumulation capacity of Cr in mycelium and highly efficient assisting *Pinus thunbergii* for phytoremediation, Chemosphere. 224 (2019) 862–872. doi: 10.1016/j.chemosphere.2019.03.015 30852466

[16] Feng M., Yin H., Peng H., Lu G., Liu Z., Dang Z., iTRAQ-based proteomic profiling of *Pycnoporus sanguineus* in response to co-existed tetrabromobisphenol A (TBBPA) and hexavalent chromium, Environ. Pollut. 242 (2018) 1758–1767. doi: 10.1016/j.envpol.2018.07.093 30061077

[17] Clemens S., Evolution and function of phytochelatin synthases, J. Plant Physiol. 163 (2006) 319–332. doi: 10.1016/j.jplph.2005.11.010 16384624

[18] Narayani M., Shetty K.V., Chromium-resistant bacteria and their environmental condition for hexavalent chromium removal: a review, Crit. Rev. Env. Sci. Tec. 43 (2013) 955–1009.

[19] Yao Y., Hu L., Li S., Zeng Q., Zhong H., He Z., Exploration on the bioreduction mechanisms of Cr(VI) and Hg(II) by a newly isolated bacterial strain *Pseudomonas umsongensis* CY-1, Ecotoxicol. Environ. Saf. 201 (2020) 110850. doi: 10.1016/j.ecoenv.2020.110850 32531571

[20] Bellion M., Courbot M., Jacob C., Blaudez D., Chalot M., Extracellular and cellular mechanisms sustaining metal tolerance in ectomycorrhizal fungi, FEMS Microbiol. Lett. 254 (2006) 173–181. doi: 10.1111/j.1574-6968.2005.00044.x 16445743

[21] Johnson A.J., Veljanoski F., O’Doherty P.J., Zaman M.S., Petersingham G., Bailey T.D., et al., Revelation of molecular basis for chromium toxicity by phenotypes of *Saccharomyces cerevisiae* gene deletion mutants, Metallomics. 8 (2016) 542–550. doi: 10.1039/c6mt00039h 27146641

[22] Flores-Alvarez L.J., Corrales-Escobosa A.R., Cortés-Penagos C., Martínez-Pacheco M., Wrobel-Zasada K., Wrobel-Kaczmarczyk K., et al., The *Neurospora crassa chr-1* gene is up-regulated by chromate and its encoded CHR-1 protein causes chromate sensitivity and chromium accumulation, Curr. Genet. 58 (2012) 281–290. doi: 10.1007/s00294-012-0383-5 23085746

[23] Li Y., Wang H., Wu P., Yu L., Rehman S., Wang J., et al., Bioreduction of hexavalent chromium on goethite in the presence of *Pseudomonas aeruginosa*, Environ. Pollut. 265 (2020) 114765. doi: 10.1016/j.envpol.2020.114765 32454358

[24] Gong D., Ye F., Pang C., Lu Z., Shang C., Isolation and characterization of *Pseudomonas* sp. Cr13 and its application in removal of heavy metal chromium, Curr. Microbiol. 77 (2020) 3661–3670. doi: 10.1007/s00284-020-02162-5 32797267

[25] Zhang J.K., Wang Z.H., Ye Y., Heavy metal resistances and chromium removal of a novel Cr(VI)-reducing *Pseudomonad* strain isolated from circulating cooling water of iron and steel plant, Appl. Biochem. Biotechnol. 180 (2016) 1328–1344. doi: 10.1007/s12010-016-2170-0 27350052

[26] Shang C., Bi G., Yuan Z., Wang Z., Alam M.A., Xie J., Discovery of genes for production of biofuels through transcriptome sequencing of *Dunaliella parva*, Algal Res. 13 (2016) 318–326.

[27] Tjaden B., De novo assembly of bacterial transcriptomes from RNA-seq data, Genome Biol. 16 (2015) 1. doi: 10.1186/s13059-014-0572-2 25583448 PMC4316799

[28] T. UniProt Consortium, UniProt: the universal protein knowledgebase, Nucleic Acids Res. 46 (2018) 2699. doi: 10.1093/nar/gky092 29425356 PMC5861450

[29] Punchi Hewage A.N.D., Fontenot L., Guidry J., Weldeghiorghis T., Mehta A.K., Donnarumma F., et al., Mobilization of iron stored in bacterioferritin is required for metabolic homeostasis in *Pseudomonas aeruginosa*, Pathogens. 9 (2020) 980. doi: 10.3390/pathogens9120980 33255203 PMC7760384

[30] Soldano A., Yao H., Punchi Hewage A.N.D., Meraz K., Annor-Gyamfi J.K., Bunce R.A., et al., Small molecule inhibitors of the bacterioferritin (BfrB)-ferredoxin (Bfd) complex kill biofilm-embedded *Pseudomonas* aeruginosa cells. ACS Infect. Dis. 7 (2021) 123–140. doi: 10.1021/acsinfecdis.0c00669 33269912 PMC7802073

[31] Lu P., Heineke M.H., Koul A., Andries K., Cook G.M., Lill H., et al., The cytochrome bd-type quinol oxidase is important for survival of *Mycobacterium smegmatis* under peroxide and antibiotic-induced stress, Sci. Rep. 5 (2015) 10333. doi: 10.1038/srep10333 26015371 PMC4450806

[32] Zhang X., Wu W., Virgo N., Zou L., Liu P., Li X., Global transcriptome analysis of hexavalent chromium stress responses in *Staphylococcus aureus* LZ-01, Ecotoxicology. 23 (2014) 1534–1545. doi: 10.1007/s10646-014-1294-7 25086489

[33] Lovley D.R., Phillips E.J., Reduction of chromate by *Desulfovibrio vulgaris* and its c(3) cytochrome, Appl. Environ. Microbiol. 60 (1994) 726–728. doi: 10.1128/aem.60.2.726-728.1994 16349200 PMC201373

[34] Magnuson T.S., Swenson M.W., Paszczynski A.J., Deobald L.A., Kerk D., Cummings D.E., Proteogenomic and functional analysis of chromate reduction in *Acidiphilium cryptum* JF-5, an Fe(III)-respiring acidophile, Biometals. 23 (2010) 1129–1138. doi: 10.1007/s10534-010-9360-y 20593301

[35] Argüello J.M., Eren E., González-Guerrero M., The structure and function of heavy metal transport P1B-ATPases. Biometals. 20 (2007) 233–248. doi: 10.1007/s10534-006-9055-6 17219055

[36] Hu P., Brodie E.L., Suzuki Y., McAdams H.H., Andersen G.L., Whole-genome transcriptional analysis of heavy metal stresses in *Caulobacter crescentus*, J. Bacteriol. 187 (2005) 8437–8449. doi: 10.1128/JB.187.24.8437-8449.2005 16321948 PMC1317002

[37] Habig W.H., Pabst M.J., Jakoby W.B., Glutathione S-transferases. The first enzymatic step in mercapturic acid formation, J, Biol, Chem. 249 (1974) 7130–7139. 4436300

[38] Liu X., Zhou M., Yang Y., Wu J., Peng Q., Overexpression of Cu-Zn SOD in *Brucella abortus* suppresses bacterial intracellular replication via down-regulation of Sar1 activity, Oncotarget. 9 (2018) 9596–9607. doi: 10.18632/oncotarget.24073 29515756 PMC5839387

[39] Chourey K., Wei W., Wan X.F., Thompson D.K., Transcriptome analysis reveals response regulator SO2426-mediated gene expression in *Shewanella oneidensis* MR-1 under chromate challenge, BMC Genomics. 9 (2008) 395. doi: 10.1186/1471-2164-9-395 18718017 PMC2535785

[40] Meng X., Hong L., Yang T.T., Liu Y., Jiao T., Chu X.H., et al., Transcriptome-wide identification of differentially expressed genes in *Procambarus clarkii* in response to chromium challenge, Fish Shellfish Immunol. 87 (2019) 43–50. doi: 10.1016/j.fsi.2018.12.055 30590169

[41] Islinger M., Li K.W., Seitz J., Völkl A., Lüers G.H., Hitchhiking of Cu/Zn superoxide dismutase to peroxisomes—evidence for a natural piggyback import mechanism in mammals, Traffic. 10 (2009) 1711–1721. doi: 10.1111/j.1600-0854.2009.00966.x 19686298

[42] Yeom D.H., Im S.J., Kim S.K., Lee J.H., Activation of multiple transcriptional regulators by growth restriction in *Pseudomonas aeruginosa*, Mol. Cells 37 (2014) 480–486. doi: 10.14348/molcells.2014.0105 24938225 PMC4086342

[43] Palma M., Zurita J., Ferreras J.A., Worgall S., Larone D.H., Shi L., et al., *Pseudomonas aeruginosa* SoxR does not conform to the archetypal paradigm for SoxR-dependent regulation of the bacterial oxidative stress adaptive response, Infect. Immun. 73 (2005) 2958–2966. doi: 10.1128/IAI.73.5.2958-2966.2005 15845502 PMC1087365

[44] Gupta P., Rani R., Chandra A., Kumar V., Potential applications of *Pseudomonas* sp. (strain CPSB21) to ameliorate Cr^6+^ stress and phytoremediation of tannery effluent contaminated agricultural soils, Sci. Rep. 8 (2018) 4860. doi: 10.1038/s41598-018-23322-5 29559691 PMC5861048

[45] Shi L., Xue J., Liu B., Dong P., Wen Z., Shen Z., et al., Hydrogen ions and organic acids secreted by ectomycorrhizal fungi, *Pisolithus* sp1, are involved in the efficient removal of hexavalent chromium from waste water, Ecotoxicol. Environ. Saf. 161 (2018) 430–436. doi: 10.1016/j.ecoenv.2018.06.004 29908454

[46] Shoaib A., Nisar Z., Nafisa A. Javaid S. Khurshid S. Javed, Necrotrophic fungus *Macrophomina phaseolina* tolerates chromium stress through regulating antioxidant enzymes and genes expression (MSN1 and MT), Environ. Sci. Pollut. Res. Int. 26 (2019) 12446–12458. doi: 10.1007/s11356-019-04457-y 30847809

[47] Wang D., Chen W., Huang S., He Y., Liu X., Hu Q., et al., Structural basis of Zn(II) induced metal detoxification and antibiotic resistance by histidine kinase CzcS in *Pseudomonas aeruginosa*, PLoS Pathog. 13 (2017) e1006533. doi: 10.1371/journal.ppat.1006533 28732057 PMC5540610

[48] Chen D., Zhao Y., Qiu Y., Xiao L., He H., Zheng D., et al., CusS-CusR two-component system mediates tigecycline resistance in carbapenem-resistant *Klebsiella pneumoniae*, Front Microbiol. 10 (2020) 3159. doi: 10.3389/fmicb.2019.03159 32047485 PMC6997431

[49] Lee S.W., Glickmann E., Cooksey D.A., Chromosomal locus for cadmium resistance in *Pseudomonas putida* consisting of a cadmium-transporting ATPase and a MerR family response regulator, Appl. Environ. Microbiol. 67 (2001) 1437–1444. doi: 10.1128/AEM.67.4.1437-1444.2001 11282588 PMC92752

[50] Adaikkalam V., Swarup S., Molecular characterization of an operon, *cueAR*, encoding a putative P1-type ATPase and a MerR-type regulatory protein involved in copper homeostasis in *Pseudomonas putida*, Microbiology (Reading). 148 (2002) 2857–2867. doi: 10.1099/00221287-148-9-2857 12213931

